# Early Stage in the Metabolism of Aminoazo Dyes in the Liver of Rats

**DOI:** 10.1038/bjc.1963.48

**Published:** 1963-06

**Authors:** J. Dijkstra


### EARLY STAGE IN THE METABOLISM OF AMINOAZO DYES IN THE LIVER OF RATS

J. DIJKSTRA

*From the National Chemical Research Laboratory, South African Council for Scientific and Industrial Research, Pretoria, South Africa*

Received for publication April 9, 1963

QUANTITATIVE studies of the binding of various carcinogenic and non-carcinogenic aminoazo dyes to proteins (Dijkstra and Joubert, 1961 ; Dijkstra and Louw, 1962) led us to investigate the mechanism of the binding process after administration of a single dose of dye. As it has been suggested (Gelboin, Miller and Miller, 1958) that the binding to proteins takes place at the time of their synthesis in the liver, trichloroacetic acid (TCA) extracts of the liver were examined in the hope of finding the dye or its metabolites bound to soluble protein precursors.

### MATERIALS AND METHODS

*Reagents.*—4′-Hydroxy-4-dimethylaminoazobenzene (4′-OH-DAB), m.p. 201·5–203·0° C., was prepared according to Miller and Miller (1948). The other aminoazo dyes used were described by Dijkstra and Louw (1962). They were dissolved in olive oil of B.P. quality.

*Treatment of animals.*—Male albino rats (weight 220–260 g.) were fasted for 6 to 7 hours before administration by stomach tube of 50 mg. of aminoazo dye in 2 ml. of olive oil. In order to avoid any possible diurnal variation in the metabolic activity of the rats at the time of dosing, the dye was always administered between 9.00 and 10.00 p.m. After dosing, the animals were fed *ad libitum* on stock diet (Dijkstra and Joubert, 1961) and tap water. At intervals the livers were removed from the animals after decapitation and thorough bleeding.

*Preparation of TCA extracts.*—The livers were minced with scissors and homogenized at 0° C. in 2 ml. of 10 per cent TCA per g. of liver, using a glass homogenizer with motor-driven perspex pestle. After standing for 10–15 minutes, the homogenate was centrifuged for 10 minutes at 1200 g at 3° C. The supernatant (*ca.* 1·8 ml.) was centrifuged a second time in order to remove all sedimentable matter. The residue of the TCA extraction was extracted repeatedly with 2·0 ml. of 7 per cent TCA per g. of liver, and the supernatants (*ca.* 2·0 ml.) were obtained in the same way. The TCA extracts were kept under an atmosphere of nitrogen.

*Estimation of dye.*—The dye content of the TCA extract was determined spectrophotometrically at 520 m$\mu$ after the addition of an equal volume of chilled concentrated hydrochloric acid. The results are expressed as the optical density ($E_{520}^{TCA}$) of these solutions using an absorption cell with a light path of 1 cm.

Free, *i.e.* alcohol-extractable, dye was determined in the solution obtained after homogenization of 2 to 3 g. of minced liver tissue with 50 ml. of absolute ethyl alcohol, heating at 75° for 10 minutes, and centrifugation at 1200 g for 15 minutes.

In order to compare the results with the amount of dye in the first TCA extract, the level of free dye was expressed as the optical density ($E_{520}^{alc}$) at 520 m$\mu$ of a solution containing the alcohol-extractable dye from 1 g. of liver in 5·4 ml. of ethyl alcohol-7N hydrochloric acid (4 : 5) for a 1 cm. light path. These results are three and a third times larger than they would have been if expressed in the same way as previously (Dijkstra and Louw, 1962).

Blank corrections were obtained after reduction of the azo dye by addition of 1 drop of a saturated solution of stannous chloride in concentrated hydrochloric acid.

FIG. 1.—Azo dye in the trichloroacetic acid extract of liver tissue of rats given 3′-MeDAB (50 mg.) in olive oil (2 ml.).

### RESULTS

Shortly after the administration of 3′-methyl-4-dimethylaminoazobenzene (3′-MeDAB) or of 2-methyl-4-dimethylaminoazobenzene (2-MeDAB) by stomach tube to rats, azo dye appeared in the TCA extracts of the liver (Fig. 1 and 2). It reached a maximum after 5 and 6 hours, respectively, and decreased to low levels within 10 hours after dosing.

In the case of 2-MeDAB (Fig. 2), the optical density at 520 m$\mu$ of the TCA extract showed a second smaller maximum at about 30 to 40 hours, suggesting the presence of another dye metabolite, before maximum binding to proteins at 40–50 hours (Dijkstra and Louw, 1962). With 3′-MeDAB, the scatter of values between 5 and 30 hours was greater and it is not possible to distinguish two separate stages. In both cases the level of TCA-soluble dye decreased to zero after 70 to 80 hours.

In contrast to these results, no dye could be detected in the TCA extracts of the liver of rats given 4-aminoazobenzene (AB) 1 to 90 hours previously ($E_{500}^{TCA}$ at 500 m$\mu$ was less than 0·01).

The bulk of the dye in the TCA extract differed from the original dye in that it was not extractable from the TCA solution with ether. Thus, after five extractions with ether, the azo dye content of the aqueous layer was still 70 to 90 per cent of the original value, whereas when 3′-MeDAB, 2-MeDAB, 4-monomethylaminoazobenzene (MAB) or AB, dissolved in a few drops of ethyl alcohol or acetone, were added to a TCA extract of normal rat liver to give a 0·002 per cent dye solution, 99·7 per cent of the dye was removed by two extractions with ether. Furthermore, the dye in the TCA extract was also not removed with an acetone-benzene mixture, as used by Mueller and Miller (1948) toe xtract N-demethylated and hydroxy derivatives of DAB. 4′-Hydroxy-DAB added *in vitro* to the TCA extract of normal rat liver was extracted quantitatively with this solvent system.

The results in Fig. 1 and 2 are based on the first extraction of liver with TCA. If the residue of the first extraction was extracted repeatedly with 7 per cent TCA,

FIG. 2.—Azo dye in the trichloroacetic acid extract of liver tissue of rats given 2-MeDAB (50 mg.) in olive oil (2 ml.).

the concentration of dye in the subsequent extracts decreased only very slowly (Table I). It is noteworthy that this is true whether the dye concentration was high or low, suggesting that the amount of dye in the TCA extracts is independent of the solubility of the azo dye as such, and is determined by some other factor.

The amount of TCA-extractable dye may be compared with that of the free, or alcohol-extractable dye, assuming that the dyes have the same extinction coefficient. As nothing is known of the chemical nature of either dye, this assumption may not be justified, nevertheless the figures so obtained serve to give some idea of the relative amounts of dye extracted.

Table I shows that for at least 8 to 9 hours the optical density of the dye in the first TCA extract was on an average 6·0 per cent of that of the alcohol-extractable dye in the case of rats given 3′-MeDAB, and 3·5 per cent in the case of 2-MeDAB. The total amounts of TCA-soluble dye removed after ten extractions were still only 31 and 20 per cent, respectively, of the amounts of alcohol-soluble dye (about 1·8 ml. of supernatant per g. of liver was removed by decantation in the first extraction and 2·0 ml. in the subsequent extractions).

The ratio of TCA-soluble to alcohol-extractable dye does not remain constant all the time, however. After 10 hours, the TCA-extractable dye in the liver

TABLE I.—*Azo Dye in the Alcohol Extract* ($E_{520}^{alc}$) *and in Consecutive Trichloroacetic acid Extracts* ($E_{520}^{TCA}$, *n=1–10*) *of Liver Tissue of Rats given 3'MeDAB or 2-MeDAB* (*250 mg./kg. body weight*) *in Olive Oil*

| Dye | Time after dosing (hours, min.) | Alcohol extract ($E_{520}^{alc}$) | Trichloroacetic acid extracts ($E_{520}^{TCA}$) | | | | | | | | | |
|---|---|---|---|---|---|---|---|---|---|---|---|---|
| | | | 1st | 2nd | 3rd | 4th | 5th | 6th | 7th | 8th | 9th | 10th |
| 3'-MeDAB | 2·40 | 1·24 | 0·075 | 0·070 | 0·063 | | | | | | | |
| ,, | 3·40 | 0·47 | 0·030 | 0·032 | 0·030 | | | | | | | |
| ,, | 4·27 | — | 0·129 | 0·115 | 0·108 | 0·092 | 0·092 | 0·071 | 0·071 | 0·060 | 0·060 | 0·046 |
| ,, | 5·07 | 1·27 | 0·063 | 0·066 | 0·065 | | | | | | | |
| ,, | 6·03 | 2·13 | 0·121 | 0·115 | 0·109 | 0·096 | 0·094 | 0·080 | 0·078 | 0·062 | 0·072 | 0·069 |
| ,, | 7·54 | 0·91 | 0·060 | 0·053 | 0·052 | | | | | | | |
| 2-MeDAB | 3·21 | 0·74 | 0·023 | 0·023 | 0·018 | | | | | | | |
| ,, | 4·12 | 2·42 | 0·080 | 0·083 | 0·073 | 0·064 | 0·059 | 0·051 | 0·044 | 0·055 | 0·054 | 0·043 |
| ,, | 5·09 | 1·77 | — | 0·057 | 0·048 | | | | | | | |
| ,, | 6·30 | 2·36 | 0·067 | 0·055 | — | | | | | | | |
| ,, | 7·50 | 2·43 | 0·111 | 0·093 | 0·081 | 0·075 | 0·068 | 0·054 | 0·056 | 0·057 | 0·058 | 0·044 |
| ,, | 8·40 | 1·56 | 0·065 | 0·067 | 0·059 | | | | | | | |

decreased to low values, while the alcohol-extractable dye was found to reach a maximum after 20 to 40 hours (Dijkstra and Louw, 1962).

### DISCUSSION

The brief appearance of TCA-soluble azo dye in the liver of rats given aminoazo dyes represents an early stage in the metabolism of these compounds. The reactions which they are known to undergo *in vivo* are reduction, N-demethylation, hydroxylation and protein binding (Miller and Bauman, 1945 ; Miller, *et al.*, 1945 ; Mueller and Miller, 1948 ; Miller and Miller, 1953). Reduction of the azo linkage need not be considered here, since this would involve loss of the chromophore. The fact that the bulk of the TCA-soluble dye is not extractable with ether or an acetone-benzene mixture distinguishes it from the original dyes administered to the rats, from their N-demethylation products, and from their hydroxylation products. The TCA-soluble dye may therefore be considered as a hitherto unknown stage in the metabolism of aminoazo compounds.

The amount and course of appearance of the TCA-soluble dye is similar in the case of the carcinogenic 3'-MeDAB and the non-carcinogenic 2-MeDAB, both of which are strongly bound to liver proteins (Miller, *et al.*, 1949 ; Dijkstra and Louw, 1962), whereas no TCA-soluble dye appeared in the case of the non-carcinogen AB, which is only bound to a very small extent. This suggests that the formation of TCA-soluble dye is more closely associated with binding of aminoazo dyes to protein, rather than directly with the carcinogenic process. This does not, however, lessen its significance, since the protein binding has been described as a necessary requirement for the induction of liver tumours (Miller and Miller, 1955), and the same may therefore be true of the formation of TCA-soluble dye.

In considering its relation to protein binding, it may be noted that TCA-soluble dye is not simply a decomposition product of protein-bound dye, since in this case its appearance should be maximal when maximum protein binding occurs at about 40 hours after dosing. Its appearance before protein binding and its disappearance at a time when protein binding becomes appreciable suggest the possibility that the

TCA-soluble dye represents an early stage in the *in vivo* binding of aminoazo dye to protein. Alternatively, it may be attributed to side effects of a reactive azo dye metabolite, which is an intermediate in the binding process. The mechanism by which aminoazo dyes become bound to liver proteins is still largely obscure and no intermediates have been reported in the literature. A study of the nature of TCA-soluble dyes may therefore throw light on the binding mechanism.

SUMMARY

Shortly after intragastric administration of a single dose of the carcinogen 3′-MeDAB and the non-carcinogen 2-MeDAB, and before binding of these dyes to proteins occurred, the trichloroacetic acid extracts of the liver of rats contained an azo dye, which differed from the dye administered and from known metabolites in that it was not extractable from its aqueous solution with ether or an acetone-benzene mixture. The concentration of this new metabolite was maximal at 5 to 6 hours after dye administration and declined to low values by 10 hours. In contrast, no formation of trichloroacetic acid-soluble dye was observed after administration of the non-carcinogenic AB, which is weakly bound to proteins. The possibility that the acid-soluble dye represents an early stage in the binding of aminoazo dyes to proteins has been discussed.

The author is indebted to Dr. H. M. Schwartz for criticism, and to Mr. J. J. Dreyer for kindly supplying the animals used in this study.

REFERENCES

DIJKSTRA, J. AND JOUBERT, F. J.—(1961) *Brit. J. Cancer*, **15**, 168.
*Idem* AND LOUW, T. B.—(1962) *Ibid.*, **16**, 757.
GELBOIN, H. V., MILLER, J. A. AND MILLER, E. C.—(1958) *Cancer Res.*, **18**, 608.
MILLER, E. C. AND MILLER, J. A.—(1955) *J. nat. Cancer Inst.*, **15**, 1571.
*Iidem*, SAPP, R. W., AND WEBER, G. M.—(1949) *Cancer Res.*, **9**, 336.
MILLER, J. A. AND BAUMANN, C. A.—(1945) *Ibid.*, **5**, 227.
*Idem* AND MILLER, E. C.—(1948) *J. exp. Med.*, **87**, 139.—(1953) *Advanc. Cancer Res.*, **1**, 339.
*Iidem* AND BAUMANN, C. A.—(1945) *Cancer Res.*, **5**, 162.
MUELLER, G. C. AND MILLER, J. A.—(1948) *J. biol. Chem.*, **176**, 535.


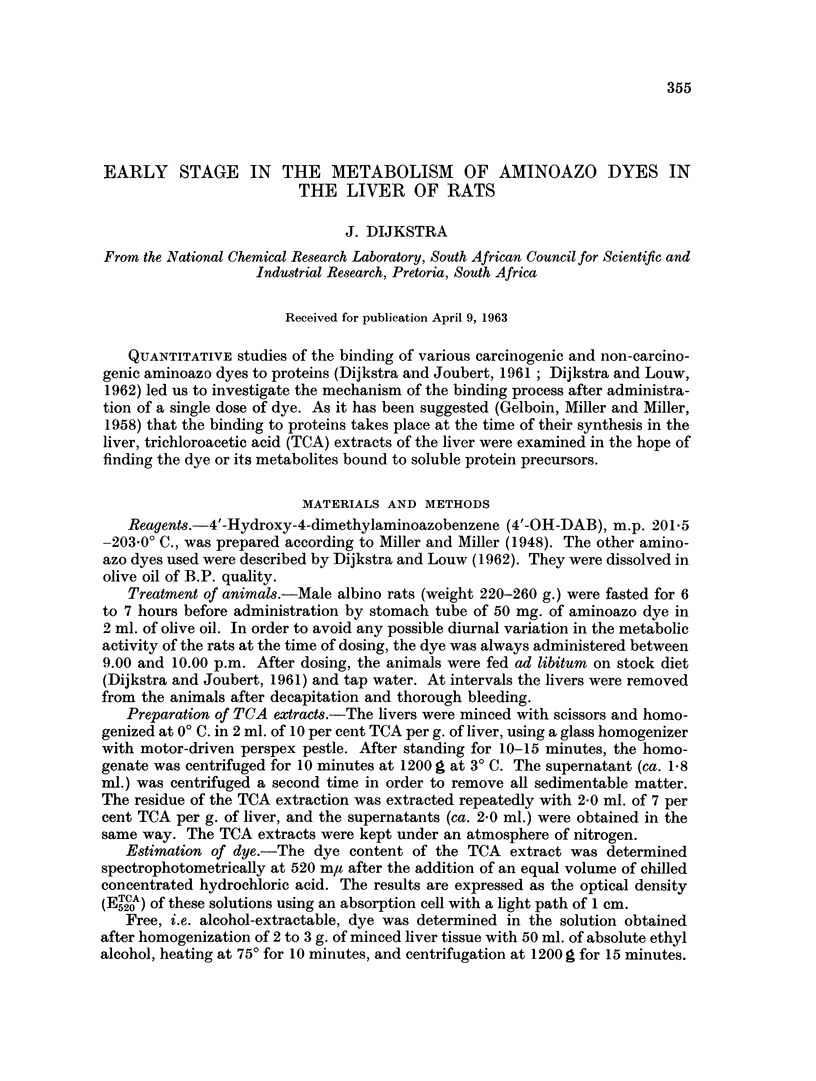

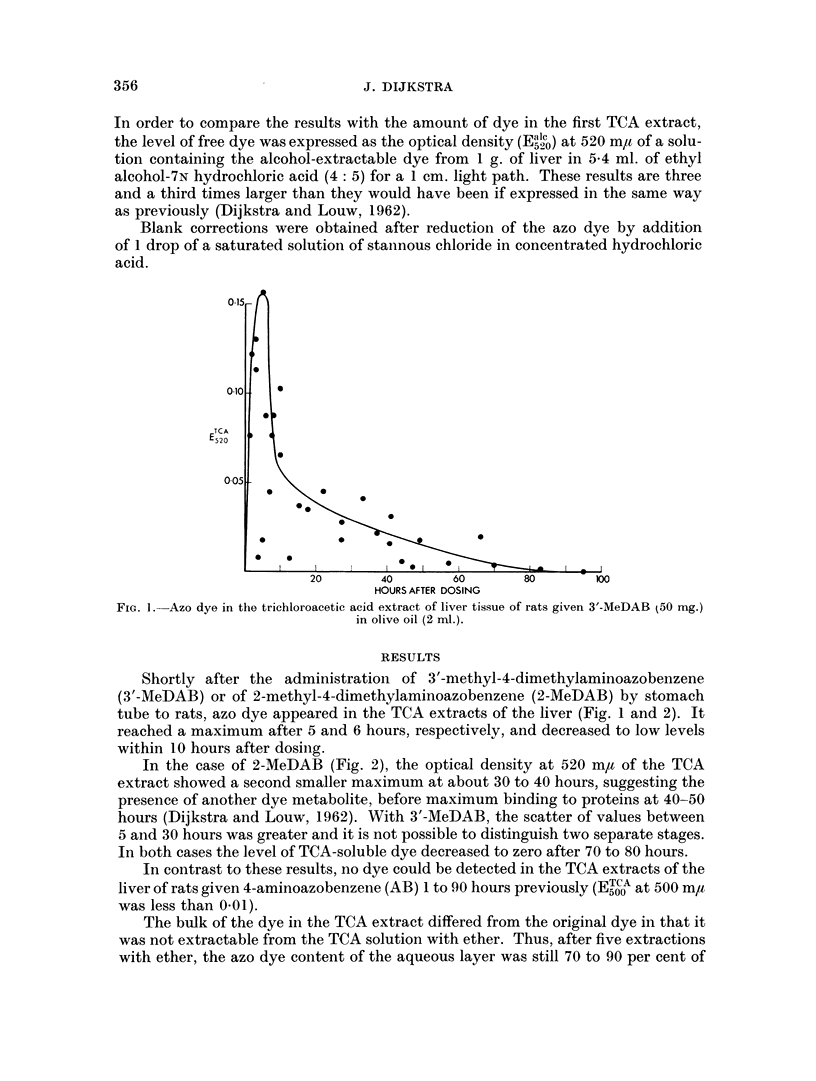

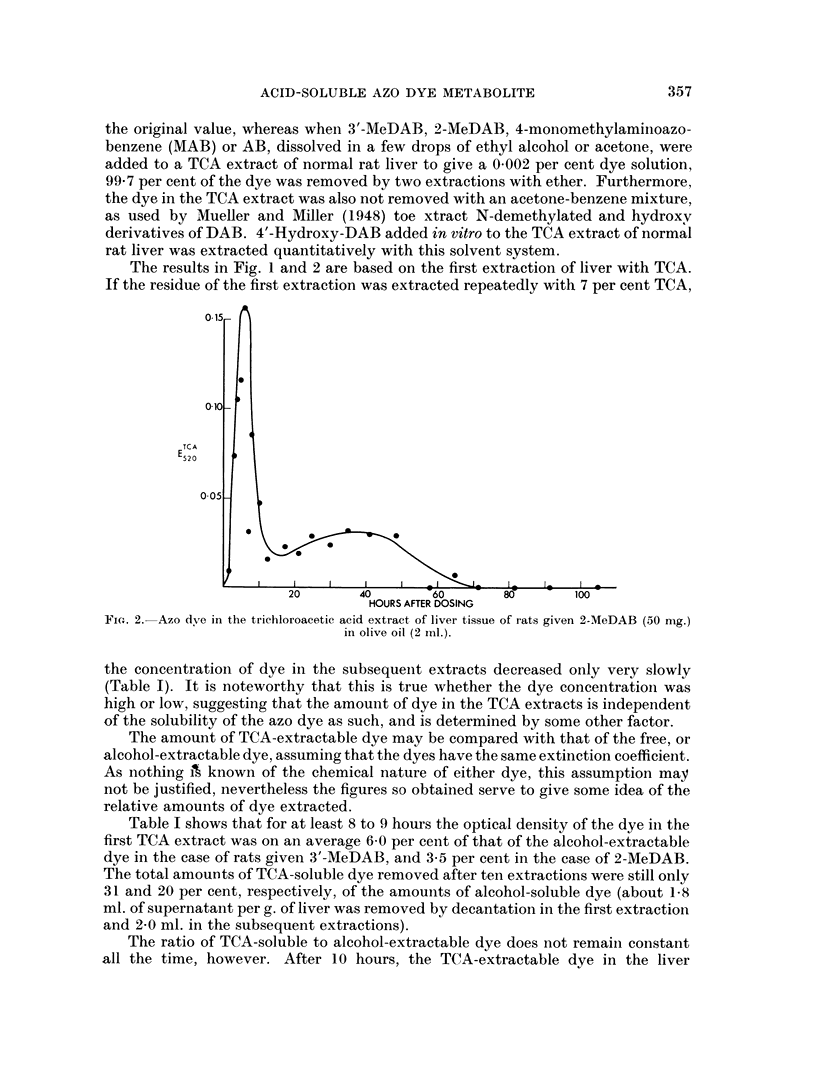

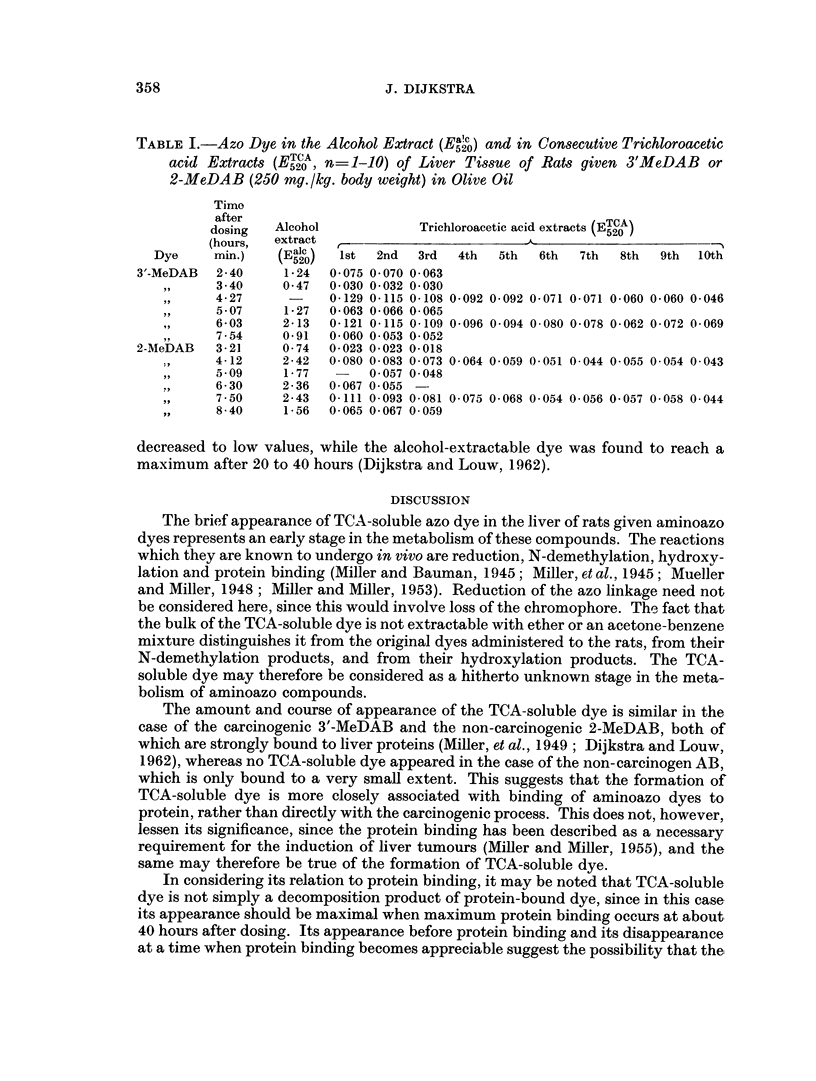

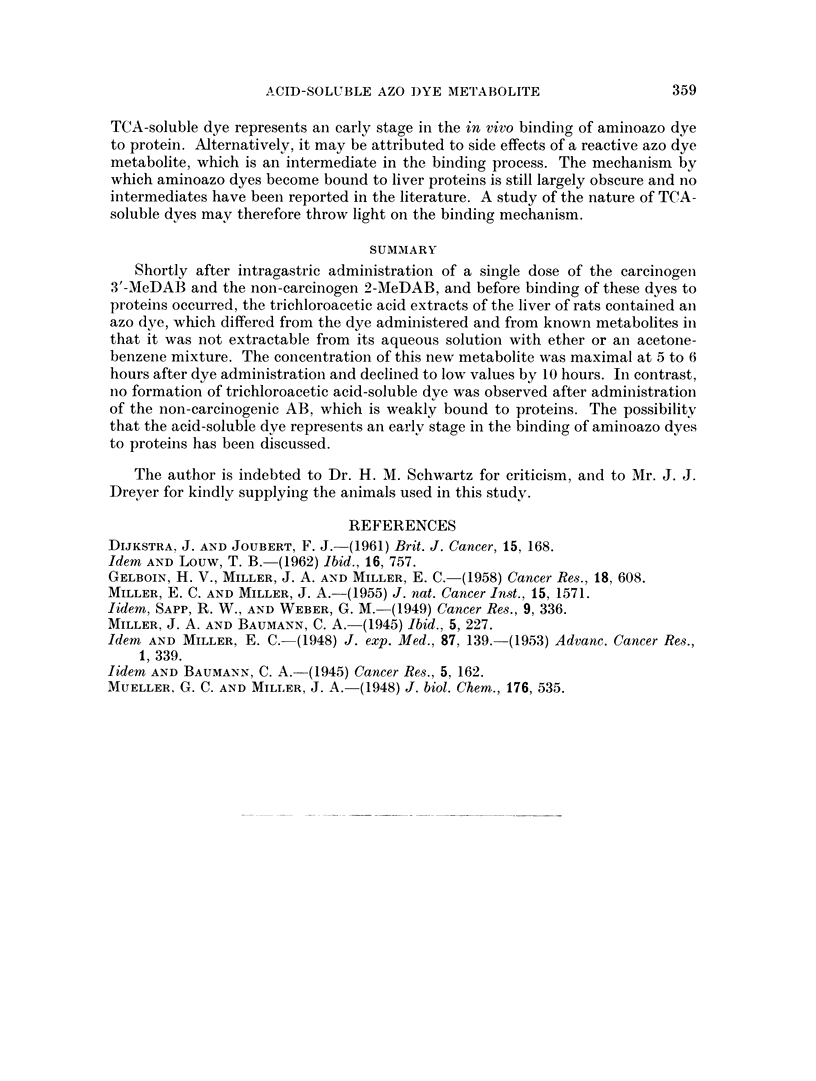


## References

[OCR_00282] DIJKSTRA J., JOUBERT F. J. (1961). Protein-bound azo dyes in the serum of rats fed 3'-methyl-4-dimethylaminoazobenzene.. Br J Cancer.

[OCR_00285] GELBOIN H. V., MILLER J. A., MILLER E. C. (1958). Studies on hepatic protein-bound dye formation in rats given single large doses of 3'methyl-4-dimethylaminoazobenzene.. Cancer Res.

